# Maximising survival by shifting the daily timing of activity

**DOI:** 10.1111/ele.13404

**Published:** 2019-10-16

**Authors:** Vincent van der Vinne, Patricia Tachinardi, Sjaak J. Riede, Jildert Akkerman, Jamey Scheepe, Serge Daan, Roelof A. Hut

**Affiliations:** ^1^ Chronobiology Unit Groningen Institute for Evolutionary Life Sciences University of Groningen Groningen 9747 AG the Netherlands; ^2^ Departamento de Fisiologia Instituto de Biociências Universidade de São Paulo São Paulo 05508 Brazil; ^3^ Present address: Department of Medicine, Integrated Research and Treatment Center for Adiposity Diseases University of Leipzig Leipzig Germany

**Keywords:** Circadian, circadian thermo‐energetics hypothesis, clock, daily energy expenditure, fitness, food restriction, foraging, nocturnal, outside enclosure, phase of entrainment

## Abstract

Maximising survival requires animals to balance the competing demands of maintaining energy balance and avoiding predation. Here, quantitative modelling shows that optimising the daily timing of activity and rest based on the encountered environmental conditions enables small mammals to maximise survival. Our model shows that nocturnality is typically beneficial when predation risk is higher during the day than during the night, but this is reversed by the energetic benefit of diurnality when food becomes scarce. Empirical testing under semi‐natural conditions revealed that the daily timing of activity and rest in mice exposed to manipulations in energy availability and perceived predation risk is in line with the model’s predictions. Low food availability and decreased perceived daytime predation risk promote diurnal activity patterns. Overall, our results identify temporal niche switching in small mammals as a strategy to maximise survival in response to environmental changes in food availability and perceived predation risk.

## Introduction

Survival of animals in the wild depends on their ability to avoid predation and obtain sufficient food to meet energetic needs (Sih 1980; McNamara & Houston 1987; Lima & Dill 1990). Acquiring food typically requires foraging away from a sheltered nesting location, resulting in elevated energetic demands and risk of predation. Animals thus face a trade‐off in which increasing foraging results in elevated predation risk (Lima & Dill 1990; Brown & Kotler 2004; Verdolin 2006). Maximising survival requires balancing this trade‐off by optimising the time spent on foraging activity and rest depending on the encountered environmental conditions. Changes in food availability or predation risk will consequently alter the optimal distribution of foraging activity and rest (McNamara & Houston 1987; Brown & Kotler 2004). Some of the most dramatic environmental changes are driven by the daily alterations between day and night (Daan 1981; Kronfeld‐Schor & Dayan 2003; Bennie *et al.* 2014). For small mammals, the night is typically associated with a reduced predation risk (Halle 1993; Moreno *et al.* 1996; Gerkema *et al.* 2013), but energetic costs are higher as a result of lower ambient temperatures (van der Vinne *et al.* 2015). To maximise survival, small mammals need to balance this trade‐off and select the daily timing of activity and rest that results in the lowest predation risk while maintaining energy balance. Most small mammals are typically nocturnal (Roll *et al.* 2006; Bennie *et al.* 2014), but shifts to diurnality are common in response to energetic challenges or increased night‐time predation (Hut *et al.* 2012; van der Vinne *et al.* 2014), suggesting temporal niche switching as a strategy to maximise survival in response to environmental changes. So far, the influence of daily activity timing on the survival of small mammals has only been considered in general (non‐quantitative) terms (Daan 1981; Lima & Dill 1990; Kronfeld‐Schor & Dayan 2003; Hut *et al.* 2012), preventing a full assessment of the implications of daily rhythms for the foraging/predation risk trade‐off. Here, we present a quantitative framework to predict the optimal temporal niche based on the encountered environmental conditions and test its predictions in mice (*Mus musculus*) living in outdoor enclosures by manipulating perceived predation risk and food availability.

## Methods

### Modelling

Energy expenditure of mice throughout the day was calculated in 10‐minute intervals using a previously described model based on the quantification of energy expenditure during the active and rest phase and at different ambient temperatures (van der Vinne *et al.* 2015). Energy expenditure could be modulated by the duration of the active phase and the selected temporal niche. Mice were presumed to maintain daily energy balance with energy intake equalling the duration of the daily active phase multiplied by the encountered foraging yield. The total daily predation risk associated with nocturnal and diurnal activity rhythms was compared for all possible combinations of foraging yields and relative daytime predation risks (daytime predation risk/night‐time predation risk) while assuming that mice maintained energy balance and predation risk was constant throughout the day and throughout the night.

### Animal studies

All procedures were approved by the Animal Experimentation Committee of the University of Groningen (DEC 5454). CBA/CaJ mice were bred in our breeding colony and subcutaneously injected with a glass‐covered passive integrated transponder (PIT) tag before being released in one of our outside enclosures. Enclosures (10 × 10 m; 53° 14′ N 06° 32′ E) were filled with white sand without vegetation while predators were kept out by a wire mesh. Each enclosure contained a hay‐filled wooden nesting box (100 × 65 × 55 cm) and an automated feeding system that provided a controlled amount of food throughout day and night. Cover between the nesting and feeding locations was provided by an overturned opaque gutter. Activity timing was determined by measuring feeder approaches using a PIT‐tag reader positioned around the feeder. The effects of alterations in energetic state and perceived predation risk were assessed by changing the daily food amount available per mouse (by doubling the food delivered or removing half the mice) and altering the available runway cover. Body temperature recordings were performed for ~ 2 months in a separate population of mice implanted intraperitoneally with Anipill temperature data loggers. All measurements were performed in both male and female mice.

An extensive description of methodological details is available in the Supporting Information.

## Results

### Optimal temporal niche depends on energetic demands and daily predation risk rhythms

**Figure 1:**
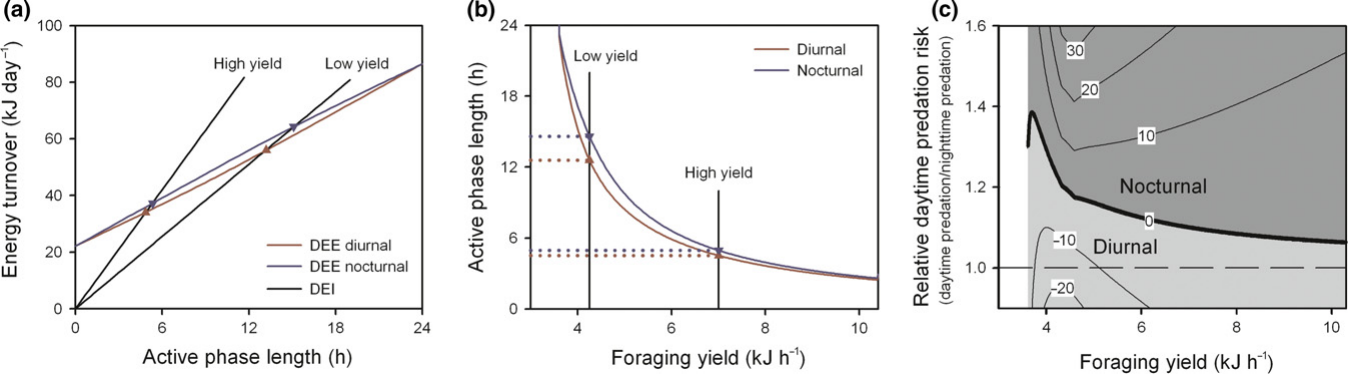
Energetic and predation risk consequences of nocturnal and diurnal activity rhythms. (a) Energetic turnover associated with nocturnal and diurnal activity rhythms. Energy balance is maintained when daily energy intake (DEI) equals daily energy expenditure (DEE). The energy turnover required to maintain energy balance for nocturnal and diurnal activity rhythms is illustrated for a low and high foraging yield. (b) The active phase length required to maintain energy balance in a nocturnal and diurnal mammal depends on the encountered average foraging yield and is shorter for diurnal activity rhythms. (c) Landscape plot of the relative difference in daily predation risk between diurnal and nocturnal activity rhythms depending on the encountered foraging yield and relative daytime predation risk. Combinations of environmental conditions where nocturnal or diurnal activity rhythms are beneficial are coloured dark or light grey, respectively. Differences in daily predation risk between nocturnal and diurnal activity rhythms are expressed as a percentage relative to the daily predation risk encountered by a nocturnal mammal with a 12 h active phase.

To maintain energy balance, daily energy intake needs to equal daily energy expenditure. Based on our previous quantification of energy expenditure during the active and resting phase in mice with nocturnal or diurnal activity rhythms (van der Vinne *et al.* 2015), daily energy expenditure was calculated for all possible active phase lengths (Fig. 1a). Daily energy intake equals the duration of the active phase multiplied by the foraging yield (Fig. 1a). Because diurnal activity rhythms reduce daily energy expenditure (Hut *et al.* 2012; Levy *et al.* 2012; van der Vinne *et al.* 2014, 2015), the duration of the active phase required to maintain energy balance is shorter for diurnal mammals at all possible foraging yields, with the greatest reduction occurring at medium to low foraging yields (Fig. 1b). This energetic benefit of diurnality is maintained in animals with crepuscular activity rhythms (Fig. S1b) and increased in energetically challenged animals (Fig. S1c) as well as when foraging yield decreases progressively during the active phase (Fig. S1d). Differences in foraging yield between day and night can counteract the energetic benefit of diurnality if foraging during the night is more efficient than daytime foraging, but conversely, elevated foraging yields during the day dramatically increase the benefit of diurnality (Fig. S1e).

Most small mammal species maintain nocturnal activity rhythms despite the energetic benefit of diurnality, presumably because of the reduced predation risk associated with night‐time foraging (Halle 1993; Moreno *et al.* 1996; Gerkema *et al.* 2013). To determine the environmental conditions at which the energetic benefit of diurnality outweighs higher daytime predation risk, survival associated with nocturnal and diurnal activity rhythms in mice maintaining energy balance was compared for all possible combinations of foraging yield and relative daytime predation risk (Fig. 1c). As expected, diurnality always increases survival when predation risk is lower during the day than the night while nocturnality is beneficial when daytime predation risk is much higher than predation risk during the night. Interestingly, in environments where predation risk was moderately higher during the day (1–1.5 times), this higher daytime predation risk is compensated by the reduction in activity duration associated with diurnality (Fig. 1c); an effect strengthened by the greater energetic benefit of diurnality at low foraging yields (Fig. 1b). All of these effects are independent of the absolute predation risk (Fig. S1f). The optimal temporal niche thus depends on the foraging effort required to maintain energy balance and the relative difference between day‐ and night‐time predation risk.

### Mice living in outdoor enclosures modify temporal niche to maximise survival

Based on the quantitative models presented above, we predict that small mammals will strive to maximise survival by becoming diurnal in response to energetic challenges and reductions in relative daytime predation risk. To test these predictions, we assessed how daily feeding rhythms of mice exposed to a temperate climate living in outdoor enclosures were affected by manipulations of food availability and cover from perceived aerial predation (Fig. S2).

**Figure 2:**
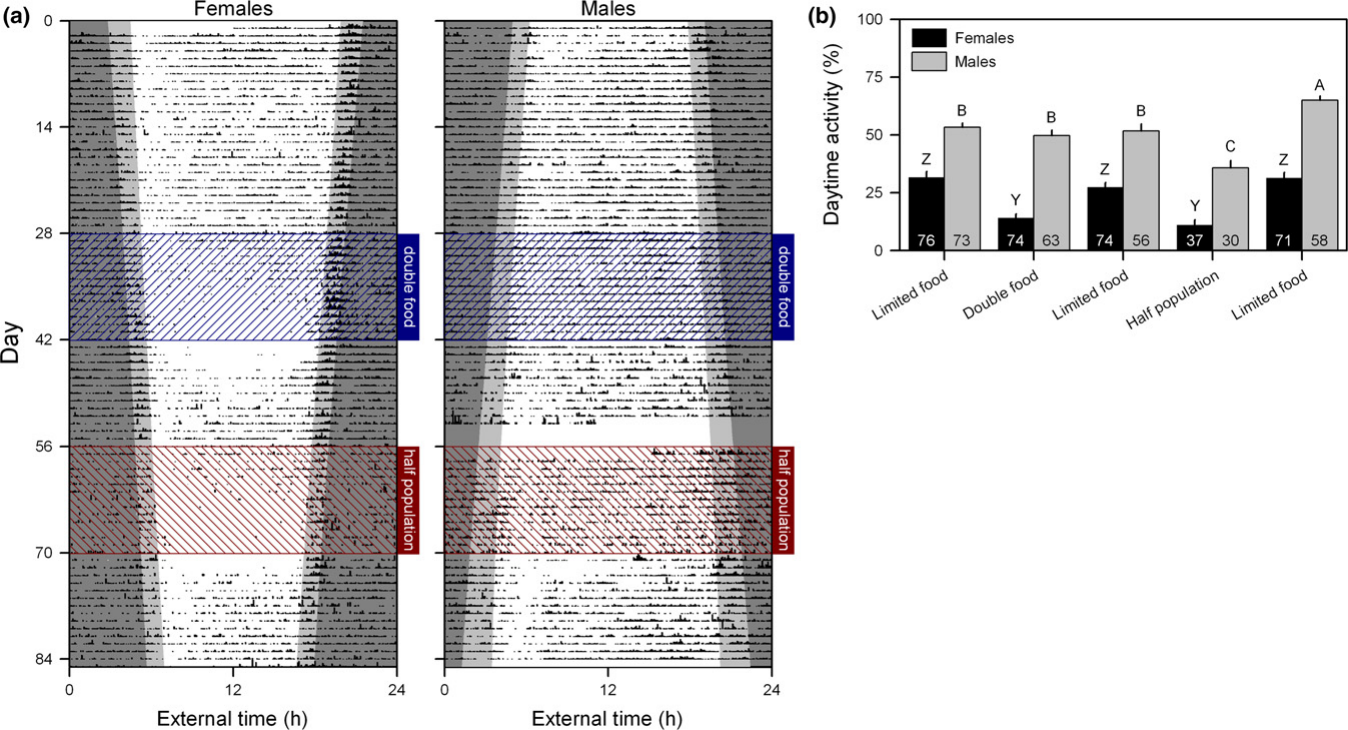
Diurnality in energetically challenged mice is reversed by increased food availability. (a) Representative actograms showing the timing of feeder visits of a female and a male population of mice housed in outdoor enclosures. Mice were maintained under a baseline condition of energy scarcity (~ 50% of *ad libitum* food intake) with food being delivered through an automated feeding system throughout day and night. Food availability was increased experimentally by doubling the delivered food amount or removing half the mice from a population. Twilight (between sunrise/sunset and nautical twilight) is indicated by the light grey background. Dark grey and white backgrounds represent night and day, respectively. Day 1 is 6 August 2014 for females and 12 March 2015 for males. (b) The percentage of feeder visits occurring during daytime was significantly reduced by increased food availability in both female and male mice. In both females and males, bars indicated by different letters are significantly different. Sample size is indicated at the base of each bar.

First, mice were exposed to chronic food restriction. Low food amounts provided evenly throughout day and night, resulted in high levels of daytime activity (Figs. 2, S3, S4 and S5). When food restriction was interrupted by 14 days of increased food availability to each enclosure (by doubling food or halving the population), mice reduced daytime activity by shifting their active phase to the night (females: *F*_4,254.5_ = 34.01, *P* < 0.0001; males: *F*_4,210.6_ = 33.09, *P* < 0.0001; Figs. 2b and S5e). Shifts in daily activity rhythms were most likely driven by changes in the energetic state of the mice, since it took ~ 1 week to respond to changes in food amount (Fig. S5f), daytime activity was negatively correlated with body mass (females: *F*_1,30.68_ = 7.906, *P* = 0.0085; males: *F*_1,6.72_ = 6.633, *P* = 0.0380; Fig. S5d), and males that died during the experiment were significantly more active during daytime in the first period of food restriction (*F*_1,72.86_ = 7.516, *P* = 0.0077; Fig. S5b).

**Figure 3:**
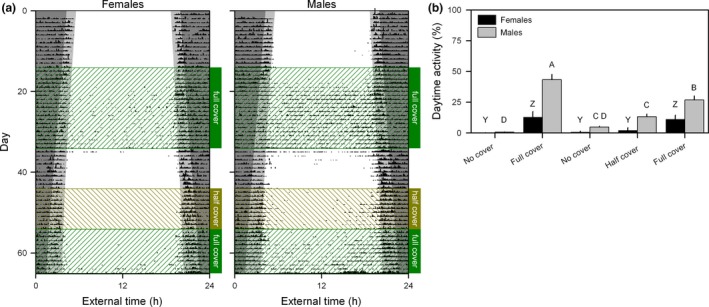
Reducing perceived predation risk increases daytime activity. (a) Population actograms showing the timing of feeder visits of female and male mice housed in outdoor enclosures exposed to changes in cover availability. Twilight (between sunrise/sunset and nautical twilight) is indicated by the light grey background. Dark grey and white backgrounds represent night and day, respectively. Day 1 is 3 April 2014. (b) The percentage of feeder visits occurring during daytime was significantly increased by runway cover in both female and male mice. In both females and males, bars indicated by different letters are significantly different.

Second, perceived aerial predation risk was manipulated by changing the amount of runway cover between the feeding and nesting location. The absence of cover dramatically reduced activity during daytime in both females and males (females: *F*_4,36_ = 13.05, *P* < 0.0001; males: *F*_4,56_ = 36.76, *P* < 0.0001; Figs. 3 and S6). Overall, our data shows that mice living under semi‐natural conditions adjust their daily activity rhythms in ways that would maximise survival in the wild by increasing diurnality in response to energetic challenges and reductions in relative daytime predation risk.

### Sex differences in temporal niche flexibility

**Figure 4:**
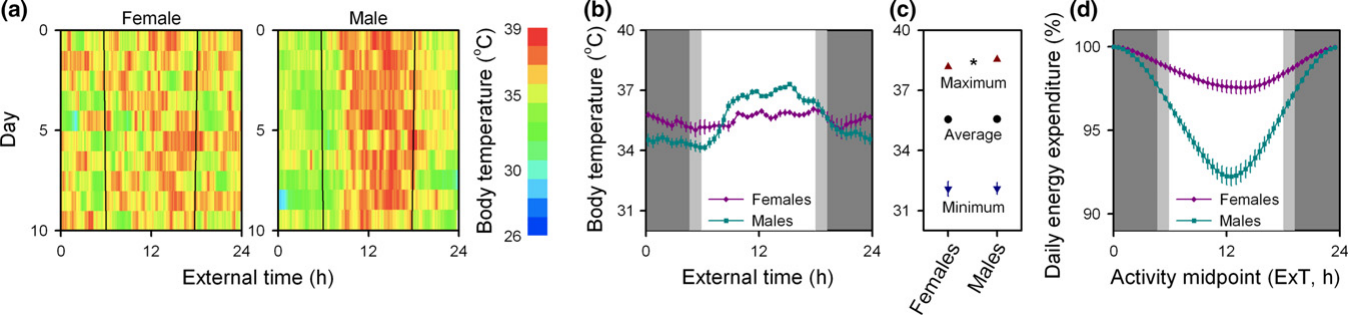
Sex differences in daily activity rhythms make diurnality more beneficial in males. (a) Representative body temperature patterns depicted as actograms in a representative female and male mouse. Day‐to‐day variability in daily activity rhythms was greater in female mice. Black lines indicate sunrise and sunset. Day 1 is 20 September 2015. (b) High day‐to‐day variability of the daily activity rhythm resulted in a depressed amplitude of the average daily body temperature rhythm in females. (c) The average, maximum and minimum body temperature measured on each individual day were mostly similar in male and female mice. (d) The energetic consequences of temporal niche switching are ~ 3 times higher in males compared to female mice.

Surprisingly, though both female and male mice responded to manipulations in food availability and perceived predation risk by altering daily activity rhythms, males were significantly more active during the day under all circumstances (Figs. 2 and 3). This observation seems to be in line with the general tendency of males to be more willing to engage in high‐risk behaviours as a way to maximise reproductive success (Clutton‐Brock & Vincent 1991; Magnhagen 1991). The higher predation risk during the day typically observed under natural conditions (Halle 1993; Moreno *et al.* 1996; Gerkema *et al.* 2013) would make elevated daytime activity levels a riskier strategy, but it might allow males to divert the energetic gains associated with diurnality towards increased reproductive investment. To further assess sex differences in daily activity rhythms, body temperature rhythms were measured in a separate cohort of female and male mice that were introduced into an established mixed‐sex population of mice. Continuous monitoring of body temperature revealed that day‐to‐day variability in daily activity rhythms of female mice was much greater compared to males (Figs. 4a and S7). This lack of day‐to‐day consistency in the daily timing of activity of females resulted in a dramatically reduced amplitude of the 10‐day average body temperature rhythm in females compared to males (*F*_47,470_ = 14.35, *P* < 0.0001; Fig. 4b), even though the average (*F*_1,10.02_ = 0.0039, *P* = 0.9514), maximum (*F*_1,9.994_ = 12.51, *P* = 0.0054) and minimum (*F*_1,10.06_ = 0.0013, *P* = 0.9719) body temperature measured on each individual day were not or only marginally different between both sexes (Fig. 4c). Importantly, the absence of a consistent daily body temperature rhythm in females dramatically reduced the energetic consequences of potential phase shifts of the activity rhythm (females: ~ 2.5%, males: ~ 8%; *F*_47,480_ = 21.49, *P* < 0.0001; Fig. 4d). Sex differences in day‐to‐day variability of daily body temperature rhythms thus provide an additional functional explanation for the consistently more diurnal activity patterns observed in male mice.

## Discussion

Our results confirm the importance of daily activity timing for the survival of small mammals under natural conditions (Daan 1981; Kronfeld‐Schor & Dayan 2003) and extend this understanding by identifying circadian flexibility as a strategy employed by mice to maximise survival depending on the encountered environmental conditions. In small mammals, diurnality is associated with reduced energetic demands (Levy *et al.* 2012; van der Vinne *et al.* 2015), although this energetic benefit needs to be balanced against typically higher predation risk during the day (Halle 1993; Moreno *et al.* 1996; Gerkema *et al.* 2013). Our quantitative assessment of this trade‐off provides a novel framework that allows integration of foraging yield and predation risk, to predict the optimal temporal niche resulting in maximal survival. Our empirical observation that mice shift their daily activity rhythm in line with our model’s predictions provides causal evidence that circadian flexibility is used by small mammals to maximise survival, as previously suggested based on field experiments (Fenn & Macdonald 1995; Bakker *et al.* 2005; Hut *et al.* 2012). The surprising observation that the energetic benefit of diurnality is ~ 3 times higher in males shows that circadian flexibility is an especially potent strategy to maximise survival in energetically challenged male mice. Given that mice are able to adjust their temporal niche within days, we feel that circadian flexibility can be an important coping strategy for small mammals exposed to frequent and rapid changes in environmental conditions. Overall, our results illustrate that daily activity rhythms are not rigid, and circadian flexibility should be considered as an important strategy to cope with rapid changes in environmental conditions such as food availability and predation risk.

## Declaration of Interests

The authors declare no conflicting interest.

## Authorship

Modelling analyses were conceived by VvdV, SD and RAH and performed by VvdV. Animal experiments were designed by VvdV, PT and RAH. Animal experiments were performed by VvdV, PT, SJR, JA and JS. VvdV performed data analyses and wrote the manuscript with input from PT and RAH.

## Data Availability

All data and code underlying modelling and behavioural analyses have been uploaded to the Figshare repository (https://doi.org/10.6084/m9.figshare.9899006).
