# Supplementary material for: Maximising survival by shifting the daily timing of activity
Source: Ecol Lett. 2019 Oct 16;22(12):2097–102. doi: 10.1111/ele.13404 (PMC6899458; doi:10.1111/ele.13404)
Supplement: Supplementary file 1 [file ELE-22-2097-s001.pdf]

Supporting Information
SI Methods

Modeling

Daily energy expenditure and daily energy intake

Daily energy expenditure of nocturnal and diurnal mice with varying activity durations was calculated using our previously published quantitative mouse energetics model based on Newtonian cooling (van der Vinne *et al.* 2015). The model calculated energy expenditure as a function of encountered ambient temperature and insulation during the active and rest phase separately using Scholander curves of *ad libitum* fed mice measured during their active and rest phase (van der Vinne *et al.* 2015). Resting animals were assumed to be huddling in groups of three in well insulated nests while energy expenditure during the active phase was increased due to higher outside chill compared to laboratory situations (van der Vinne *et al.* 2015). Animals were assumed to encounter the average September daily temperature profile (KNMI weather station, Eelde, NL; WMO #06280; 53° 07' N 06° 32' E; 2001-2010) during the active phase (van der Vinne *et al.* 2015). The buffered daily nest temperature cycle (nest buffering constant: 0.0722 h^{-1}) was derived from the outside daily temperature cycle and assumed to be encountered during the rest phase (van der Vinne *et al.* 2015). Using the adjusted Scholander curves and daily temperature cycles, energy expenditure of nocturnal and diurnal animals was calculated in 10-minute intervals. Daily energy expenditure was calculated for activity durations between 0 h and 24 h in 20-minute steps assuming consolidated active and rest phases. The active phase was centered around midnight or noon for nocturnal or diurnal animals, respectively, because these are the energetically optimal and worst phases under nearly all environmental conditions (van der Vinne *et al.* 2015). The consequences of temporal niche switching were also calculated for energetically challenged animals using Scholander curves obtained from mice challenged by the working-for-food protocol (van der Vinne *et al.* 2014), and for animals with crepuscular activity rhythms. These crepuscular rhythms extended activity throughout twilight first, followed by active-phase length extensions from sunset into the day or night for diurnal and nocturnal activity rhythms respectively. Daily energy intake was calculated by multiplying the duration of the active phase with the foraging yield. The consequences of nocturnal and diurnal activity rhythms were also calculated for animals encountering a foraging yield that declined progressively with the duration of the active phase (0.1% - 3% hourly yield decline) or differences in day- and night-time foraging yield.

Activity duration

The activity duration required to maintain energy balance was determined for nocturnal and diurnal rhythms as the activity duration for which daily energy expenditure equals daily energy intake. The required duration was determined by linearly interpolating the difference between daily energy expenditure and daily energy intake within the 20-minute intervals for which both were calculated.

Daily predation risk

The influence of day-night differences in predation risk on the total daily predation risk was assessed by varying the ratio (relative daytime predation risk) between day- and night-time predation risk. The total daily predation risk was calculated for nocturnal and diurnal mice at all possible constant foraging yields. The daily predation risk was determined by integration of the instantaneous survival rate per 10-minute interval over the full active phase. Rest phase predation

risk for a burrowing mouse was assumed to be negligible, because changes in basal (non-rhythmic) burrow predation risk do not alter the survival consequences of shifting daily activity rhythms. The calculation of the survival consequences of nocturnal and diurnal activity rhythms was performed for different night-time predation risk levels to confirm that the temporal niche influence on survival was only marginally affected by the overall predation risk level. The survival benefit of diurnality was calculated by subtracting the total daily predation risk for a nocturnal mouse from that of a diurnal mouse. The survival benefit of diurnality was calculated as a ratio relative to the daily predation risk of a mouse active for 12 h throughout the night.

Animal studies

Animals

All procedures were approved by the animal experimentation committee of the University of Groningen (DEC 5454). CBA/CaJ mice were bred in our breeding colony and maintained with same-sex littermates in a 14h:10h light-dark cycle. The mice were housed individually in a 12h:12h light-dark cycle approximately one week before release in our outside enclosures. During this week, the mice were subcutaneously injected with a glass-covered passive integrated transponder (PIT; 11.5 x 2.2 mm; Trovan ID100) tag under the skin of the back after being lightly anesthetized with isoflurane.

Semi-natural enclosures

Our four outside enclosures (10 x 10 m each) are located outside our laboratory in Groningen, the Netherlands (53° 14' N 06° 32' E). The enclosures are surrounded by a 1 m high wall and a 50 cm deep continuous underground wire mesh while predators are kept out by an overhead wire mesh. The enclosures are filled with white sand without vegetation. Nesting opportunities were provided in a hay-filled wooden box (100 x 65 x 55 cm) located in one corner of the enclosure while the feeder and *ad libitum* water were located in the opposite corner (Fig. S2). The 5 m distance between nesting location and feeder was covered for 80% by a 4 m long overturned opaque grey PVC gutter, serving as runway cover. Food (AM II diet rodent chow 10 mm, 17.3 kJ/g, Arie Blok, Woerden, the Netherlands) was provided in a feeding hopper tube, which extended from the observation cabin into the enclosure and which was automatically filled by a custom-build conveyor belt rotating 2.6 mm every 15 minutes throughout the day. Food delivery was distributed evenly over the day by spreading out the daily amount of food pellets over 25 cm on the conveyor belt. Feeding activity of the mice was registered by detecting PIT tags using an antenna surrounding the feeder and stored for all enclosures on a single data storage device (Trovan Automated PIT recording system; Dorset group BV, Aalten, the Netherlands).

Environmental conditions manipulations

The effects of changes in food availability at the population level on the daily distribution of activity were assessed in six single-sex (3 female, 3 male) populations of mice. 76 Adult female mice (age: 4 - 13 months) were released in three enclosures in early April 2014 while 83 adult male mice (age: 3 - 11 months) were released in three enclosures in early February 2015. The daily food amount was restricted to induce robust diurnal activity rhythms (females: enclosure 1: 2.33 $g \cdot mouse^{-1} \cdot day^{-1}$, 2: 1.86 $g \cdot mouse^{-1} \cdot day^{-1}$, 3: 2.26 $g \cdot mouse^{-1} \cdot day^{-1}$; males: enclosure 1: 2.73 $g \cdot mouse^{-1} \cdot day^{-1}$, 2: 3.09 $g \cdot mouse^{-1} \cdot day^{-1}$, 3: 2.71 $g \cdot mouse^{-1} \cdot day^{-1}$). The role of the energetic state in shaping the daily distribution of activity was assessed in each of the enclosures during a three-month period by 14-day manipulations. The manipulations consisted of either a doubling of the

daily food amount (double food) or the removal of half of the mice from the enclosure (half population) with periods of food limitation before, in between and following the manipulations. The population reduction was performed by catching as many mice as possible over a two-hour period. Approximately half of the mice present in the enclosure were removed aiming at equal body masses of the removed mice and the mice that remained in the enclosure.

The changes in the daily distribution of activity following manipulations in runway cover were assessed in a single mixed-sex population. A group of 15 males and 10 females (age: 3 - 11 months) was released in mid-January 2014 and maintained throughout the experiment in a single enclosure. This allowed the mice to reproduce, but no pups were observed during the experiment or in the three months following the experiments. The daily food amount was chosen as the maximal amount for which all food was eaten (4.5 $g \cdot mouse^{-1} \cdot day^{-1}$). The influence of the availability of runway covers between the nesting location and feeder was assessed over a period of 65 days starting on 3 April 2014. During this period, there was either no cover, half cover (2 x 1 m) or full cover (4 m) available between the nesting box and feeder.

Body temperature recordings

Body temperature recordings were performed with high sampling rate (1 data point / 3 min), enabling a detailed assessment of daily activity rhythms, which could not be obtained with feeder activity data. Six female and six male mice (age: 4 – 8 months) were implanted intraperitonially with temperature dataloggers (Anipill, Bodycap, Hérouville Saint-Claire, France) under isoflurane anesthesia. Loggers were inserted through a 1.5-2 cm ventral midline incision, which was sutured with polyglycolic acid threat for the peritoneum and muscle layers and tissue adhesive (Vetbond 3M, St. Paul, MN, USA) for the skin. Hypothermia was prevented during surgery by placing the mouse on a thermal blanket. After implantation, mice were individually housed for >7 days in cages placed in an outdoor facility that provided shelter from rain and direct radiation but not from outside ambient temperatures. During this recovery period, mice were provided with nesting material as well as *ad libitum* food and water access. Following recovery from surgery, mice were released in early July 2015 into an enclosure which already contained a mixed-sex population subjected to a constant daily food supply. The total daily food amount provided to the enclosure was maintained constant throughout the experiment. Because of the constant daily food amount provided to this enclosure, the population was approaching carrying capacity of the energy (food) resources. Because of new mice being born in the population the total number of mice in the enclosure at any given time was unknown. Based on the observation that all food was eaten quickly after being provided and the diurnal activity rhythms of the mice (Fig. S7), we inferred that the mice were energetically challenged. Recordings took place until the battery of the temperature loggers ran out, which occurred in mid-October.

Data analyses and statistics

The daily distribution of feeder approaches was assessed by calculating the daytime activity percentage (feeder approaches between sunrise and sunset divided by total daily feeder approaches for each individual). The influence of different treatments was assessed by comparing the daytime activity percentage during the last seven days of a treatment. Survival was estimated using recordings of feeder approaches with a mouse assumed to be dead the day after its last feeder visit. The comparison of daytime activity percentage between surviving animals and animals that died during the experiment was performed using activity data recorded during the second week of food

restriction. Body mass was recorded of all mice caught preceding the population manipulation and correlated with the daytime activity percentage during the preceding seven days.

Body temperature rhythmicity was assessed during a ten-day interval around the September equinox. Average body temperature profiles were calculated for females and males over this full ten-day interval. The daily minimum, average and maximum body temperature were determined per mouse per day, describing phase-independent daily body-temperature characteristics. The energetic consequences of temporal niche switching were calculated for each of the 12 mice individually using the energetic model with Scholander curves of energetically challenged mice described previously (van der Vinne *et al.* 2014). To determine the energetic consequences of potential phase shifts of the activity rhythm, the ten-day body temperature recording was converted to activity and rest by assuming that mice were active when body temperature was above an animal-specific threshold and in rest when body temperature was below. This threshold was set for each mouse individually so that each animal was active for 50% of the time to normalize activity levels between animals. The energetic consequences of phase-shifting the observed activity pattern were then calculated by shifting the observed activity rhythm of each mouse in 30 min steps with the phase resulting in the highest daily energy expenditure being assumed to have an activity midpoint at mid-night.

Statistical comparisons (two-sided; alpha = 0.05) were made using restricted maximum likelihood mixed effects models using animal ID and enclosure as random variables in SAS JMP 7.0, followed by Tukey HSD post-hoc comparisons when appropriate. Residuals were inspected visually to confirm the assumptions of normalcy and heterogeneity of variance. Error bars in figures represent standard error of the mean.

SI References

van der Vinne V., Riede S.J., Gorter J.A., Eijer W.G., Sellix M.T., Menaker M., *et al.* (2014) Cold and hunger induce diurnality in a nocturnal mammal. *Proc. Natl. Acad. Sci. U.S.A.* 111, 15256–15260.

van der Vinne V., Gorter J.A., Riede S.J., Hut R.A. (2015) Diurnality as an energy-saving strategy: energetic consequences of temporal niche switching in small mammals. *J. Exp. Biol.* 218, 2585–2593.

SI Figures

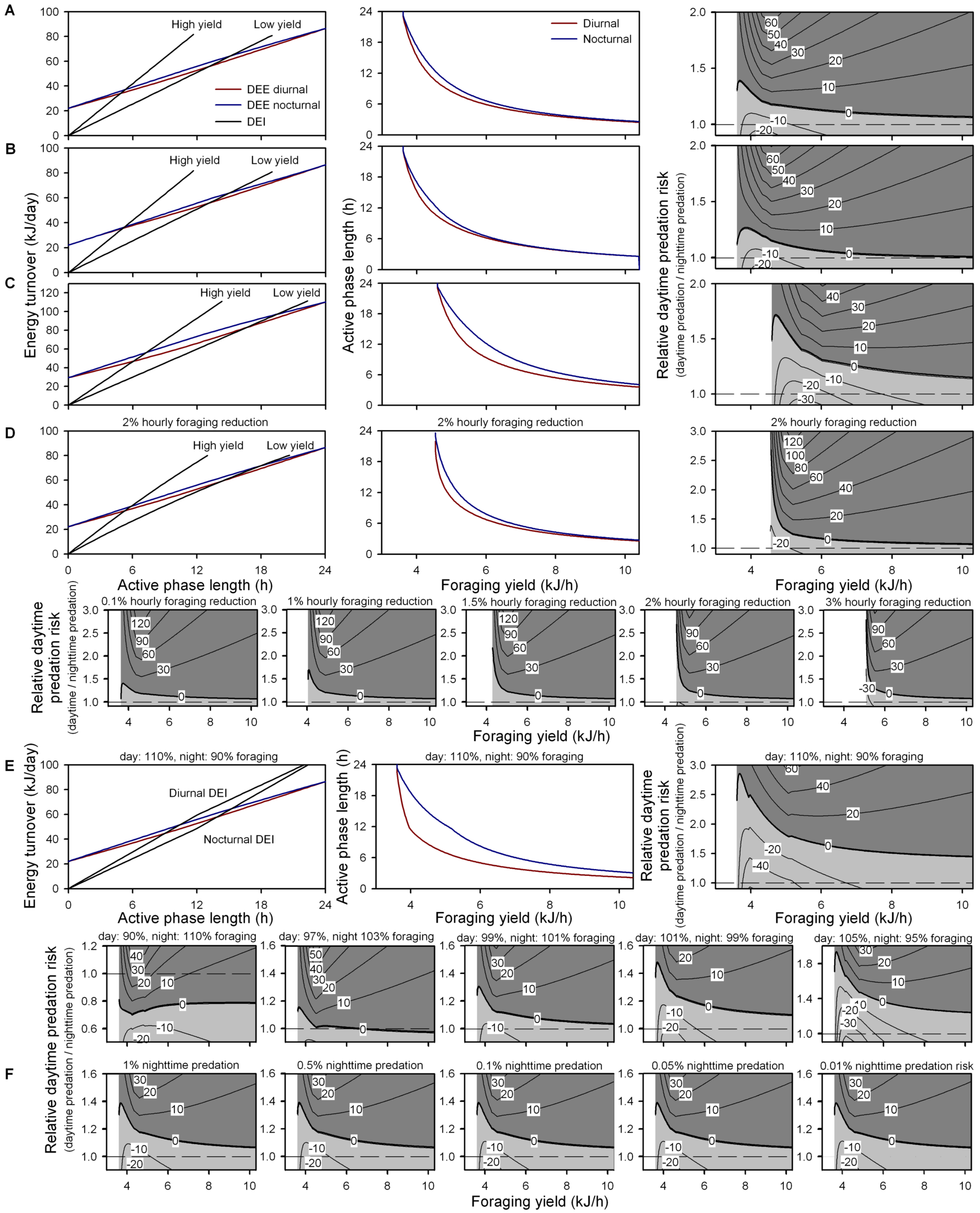

Fig. S1. Energetic and predation risk consequences of nocturnal and diurnal activity rhythms; model assumptions and validation. (*A-E*) Energy turnover [daily energy expenditure (DEE) or daily energy intake (DEI)] depends on the active phase length (left), as well as the

required active phase length to maintain energy balance at different foraging yields (middle). Daily predation risk depends on the encountered foraging yield and relative daytime predation risk (right). All of these measures are affected by the daily activity rhythm (nocturnal or diurnal). (*A*) Model outcomes for the baseline model as also presented in Figure 1. Data is replotted here for comparison purposes. (*B*) Model outcomes for a model assuming a crepuscular activity pattern with activity occurring preferentially during twilight, extending from sunset into the light or dark phase for diurnal and nocturnal activity patterns, respectively. (*C*) Model outcomes for a model incorporating Scholander curves of energetically challenged mice. Physiological changes associated with exposure to energetic challenges increase the energetic benefit of diurnality, resulting in diurnality being associated with improved survival at higher relative daytime predation risks. (*D*) Model outcomes for a model assuming a progressively declining foraging yield throughout the duration of the active phase. Different rates of foraging yield decline are assessed in the bottom five panels. Sharper declines in the foraging yield increase the survival benefit associated with diurnality, especially at very low foraging yields. (*E*) Model outcomes for a model assuming day/night differences in foraging yield. The relative survival consequences of nocturnal and diurnal activity rhythms are assessed for different day/night foraging yield rates in the lower five panels. The energetic benefit of diurnality is counteracted when day- and night foraging yields are ~97% and ~103%, respectively. (*F*) Model outcomes for different baseline predation levels. The similar outcomes of the different models illustrate that the optimal temporal niche does not dependent on the absolute hourly predation risk but depends on the relative differences in predation risk between day and night.

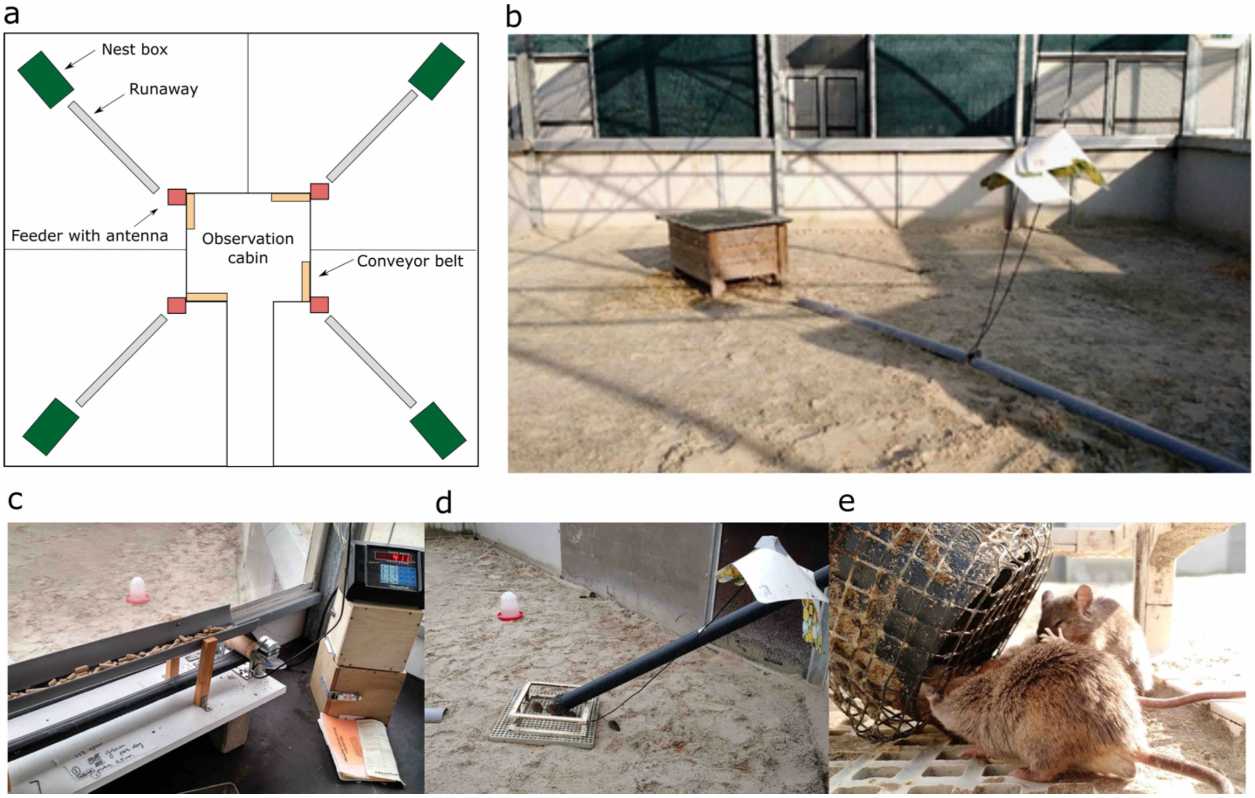

Fig. S2. Overview of semi-natural enclosures used in study. Schematic depiction (*A*) and photographs of the four enclosures used. Each enclosure (10 x 10 m) contained a hay-filled wooden box that provided nesting opportunities (*B*). Runway cover connected the nesting box with the feeding location (*B*). Food was delivered throughout day and night from a clock-controlled conveyor belt (*C*) into a feeding tube extending into the enclosure (*D*). Mice could obtain food from the feeding tube through the wire-mesh covered lower opening (*E*). Feeder approaches were measured by a PIT-tag recording antenna placed around the feeding location. Water was provided *ad libitum*.

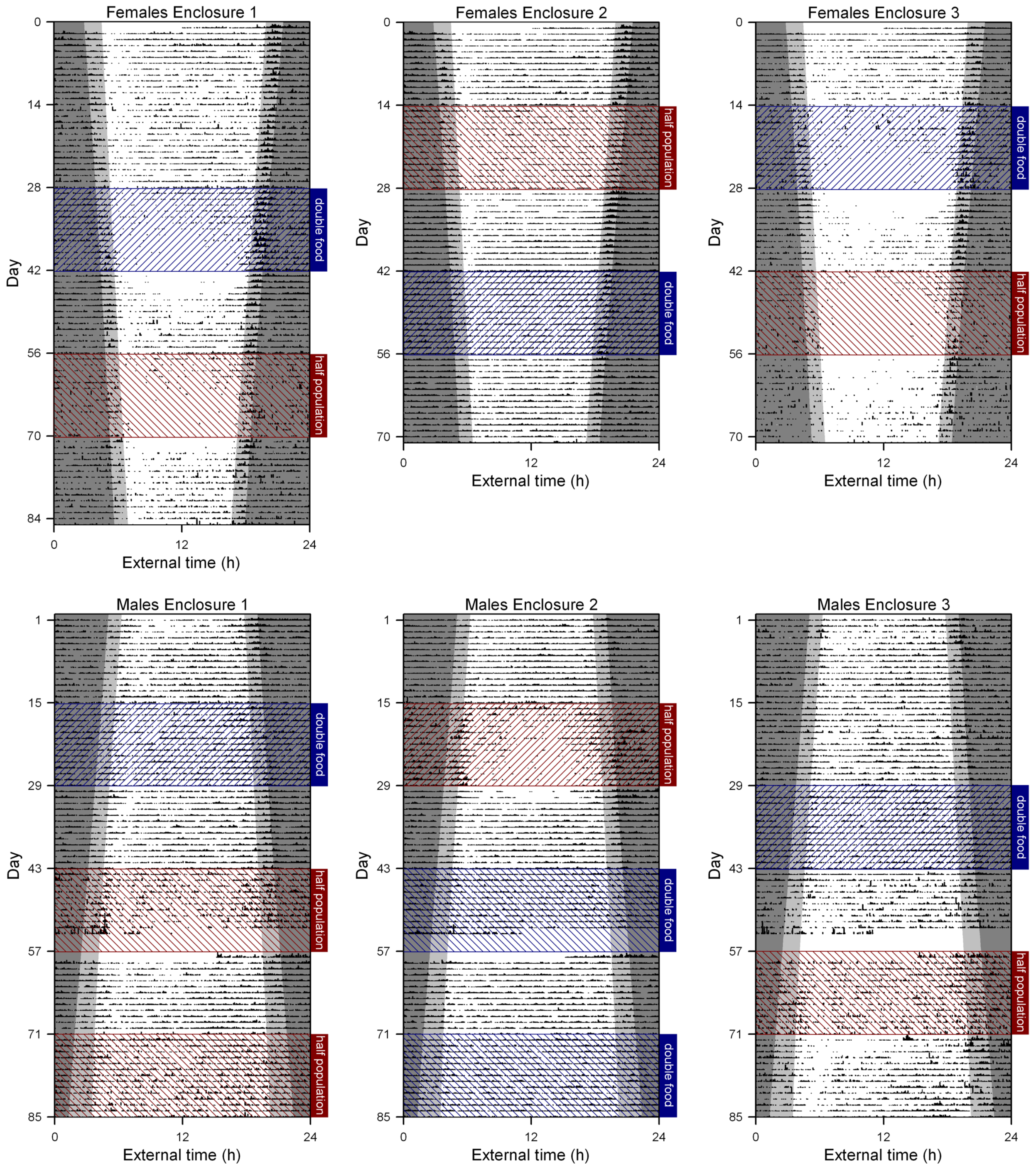

Fig. S3. Population level actograms of all 6 enclosures exposed to manipulations of food availability. Actograms representing the timing of feeder visits of 3 populations of female mice (top) and 3 populations of male mice (bottom) housed under semi-natural conditions. Mice were maintained under a baseline condition of energy scarcity (~50% of *ad libitum* food intake) with food being delivered through an automated feeding system throughout the day and night. Food availability was increased experimentally by doubling the delivered food amount or removing half

the mice from a population. Twilight (between sunrise/sunset and nautical twilight) is indicated by the light grey background. Dark-grey and white backgrounds represent night and day, respectively. Day 1 is 6 August 2014 for females and 12 March 2015 for males. Because data was not recorded during the last days of manipulation 2 in male enclosures 1 and 2, these manipulations were repeated at the end of the experiment.

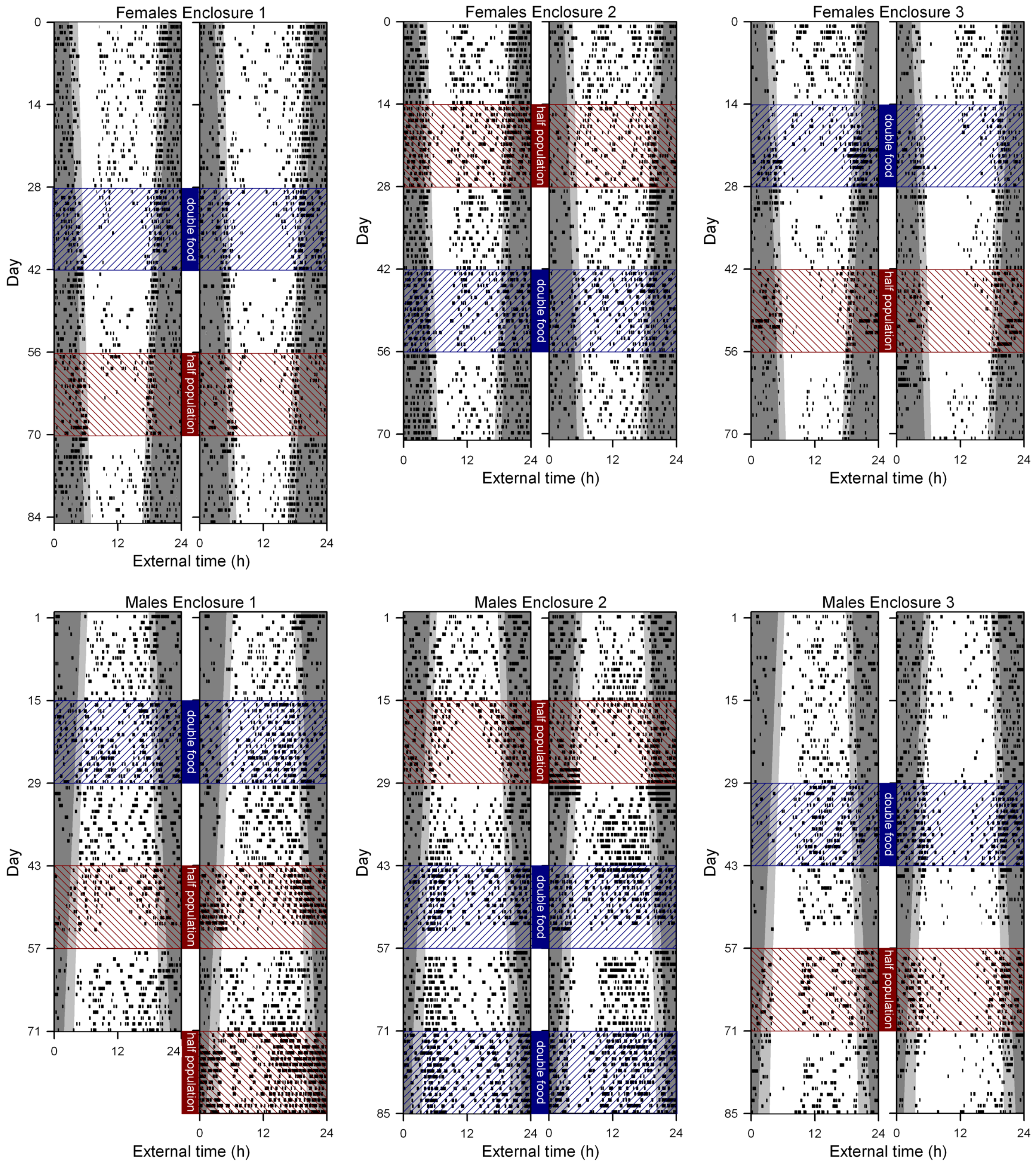

Fig. S4. Representative actograms of individual mice exposed to manipulations of food availability. Representative actograms of 2 mice per enclosure as presented in Figure S3.

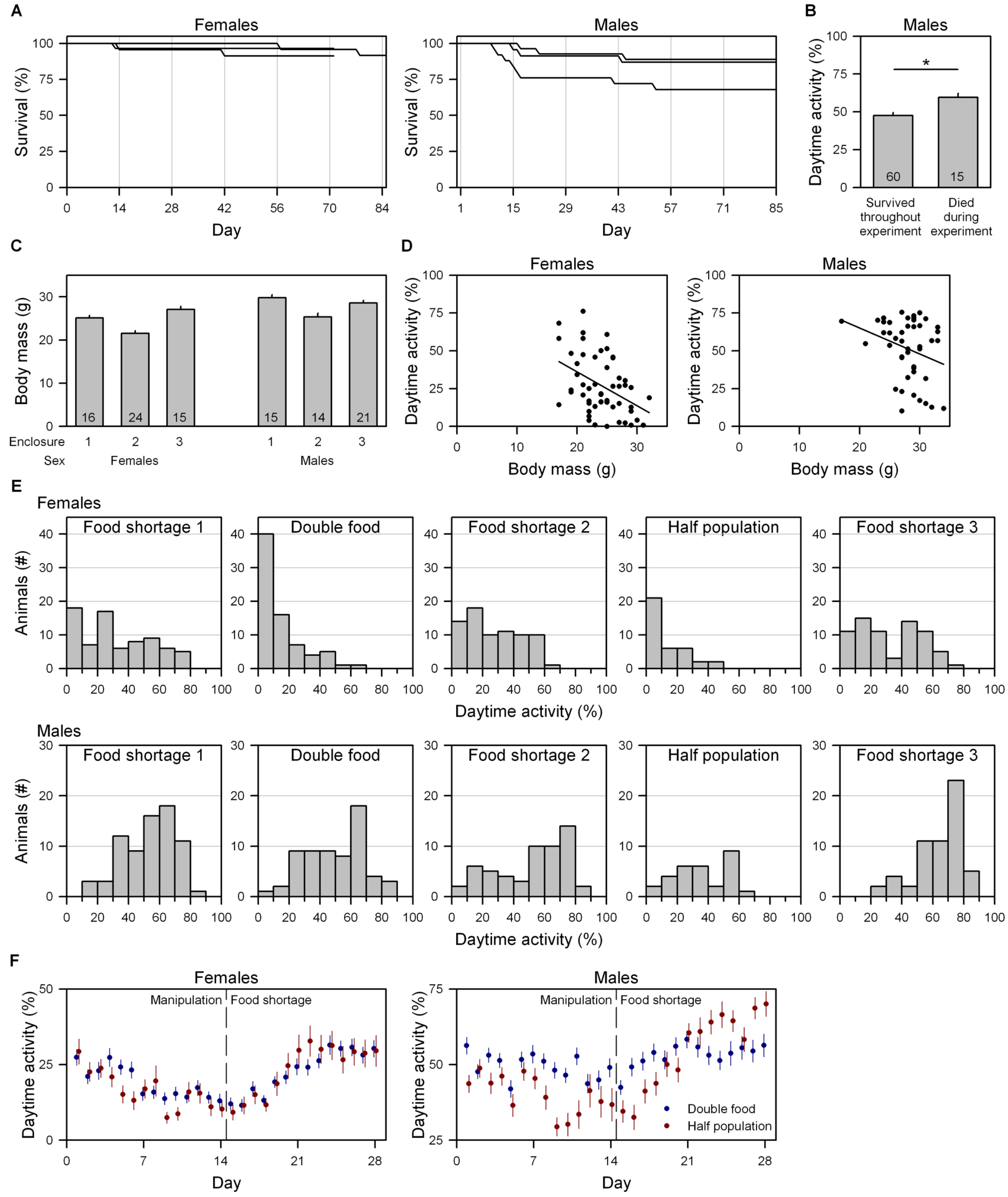

Fig. S5. Quantifications of experiments assessing effects of food availability manipulations. (*A*) Survival curves for female and male mice exposed to manipulations of food availability. Each curve corresponds to one population of mice. (*B*) The percentage of feeder visits occurring during the day was significantly higher for male mice that died at a later point during the food manipulation experiment. The daily timing of feeder visits was assessed during experimental day 8-14. Sample size is indicated at the base of each bar. This assessment was only performed in male mice because the high survival precluded a similar analysis in female mice. (*C*) Body mass of mice caught at the start of the population-size manipulation. Sample size is indicated at the base of each bar. This sample size does not represent the total number of mice in each enclosure since not all

mice were caught. (*D*) The percentage of feeder visits occurring during the day is negatively correlated with body mass. Data is presented separately for female and male mice. (*E*) Histograms depicting the distribution of daytime activity percentage during each of the 5 manipulations is presented for females (top) and males (bottom). (*F*) Daily changes in the percentage of feeder visits occurring during the day are presented for the 14-day manipulation period and the consecutive 14-day food restriction period. Daytime activity levels typically stabilize during the second 7 days of each treatment (day 8-14 and day 22-28).

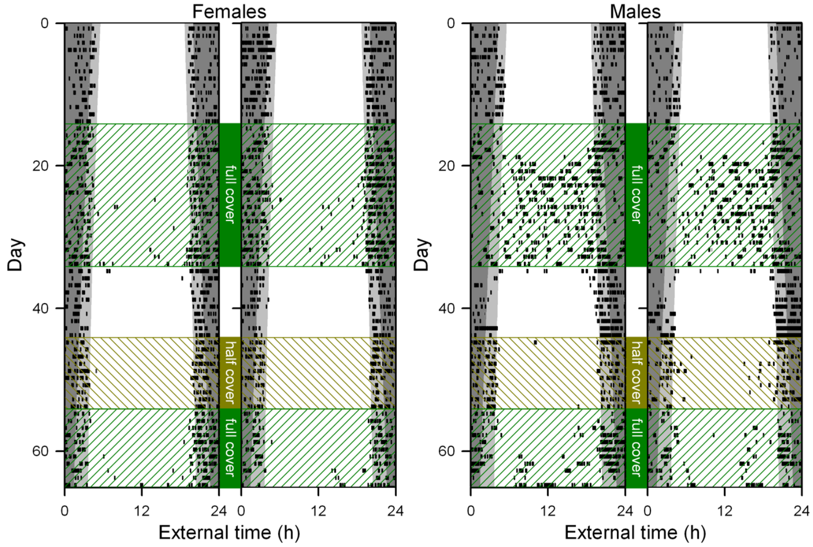

Fig. S6. Representative actograms of individual mice exposed to manipulations of cover availability. Representative actograms of 2 mice per sex exposed to manipulations in cover availability. Activity represents the timing of feeder visits. Twilight (between sunrise/sunset and nautical twilight) is indicated by the light grey background. Dark-grey and white backgrounds represent night and day, respectively. Day 1 is 3 April 2014.

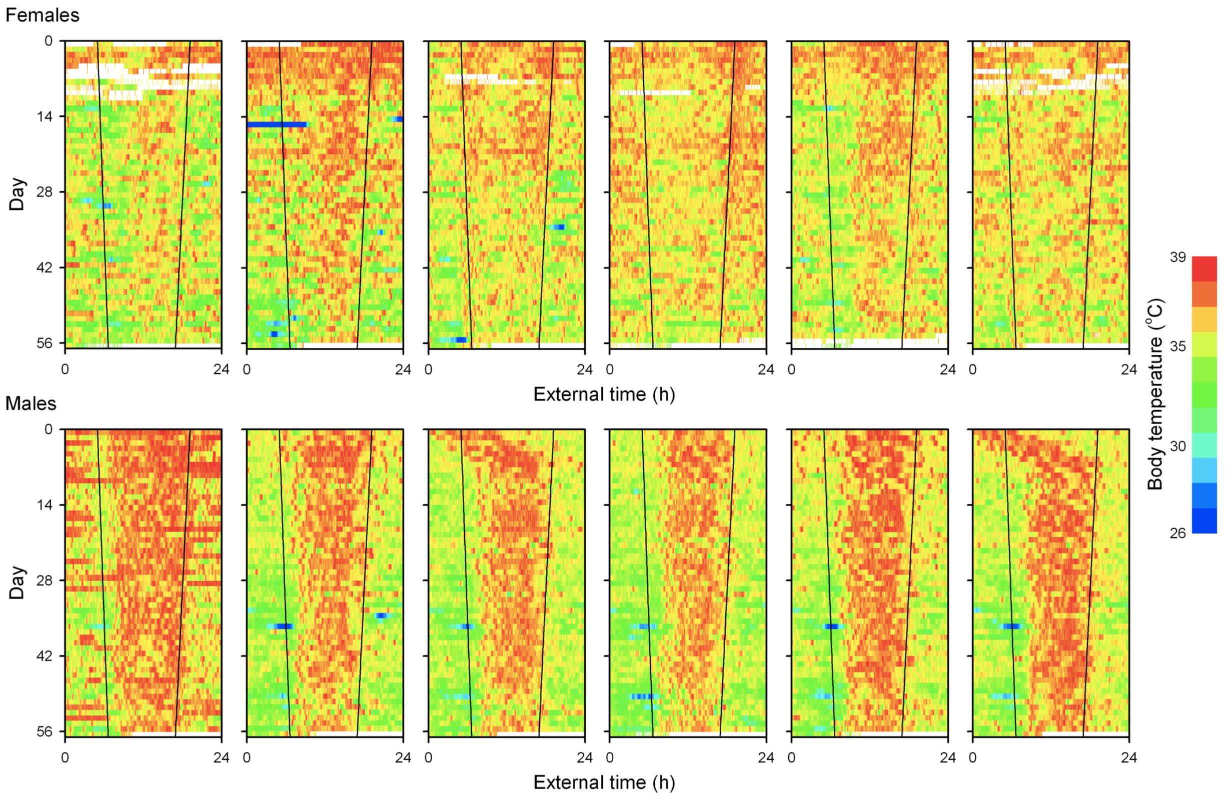

Fig. S7. Actograms depicting body temperature rhythms of 6 female (top) and 6 male (bottom) mice living under semi-natural conditions. The recorded mice were part of a larger mixed-sex population living under food restriction. Body temperature recordings were performed over a 57-day period. Sunrise and sunset are indicated by black lines. Day 1 is 25 August 2015.
